# Past and Present

**Published:** 1892-09-10

**Authors:** 


### THE DOCTOR'S ARMCHAIR.

### PAST AND PRESENT.

Is the world fighting a winning or a losing battle against epidemics, common diseases, and the manifold forces of evil which man finds opposed to his ease and sensuous pleasure? This is a question which, whilst it is profoundly interesting to the man of science and the philosopher, is of most practical importance to the average, every-day man and woman. The scientific man usually has sufficient science to enable him to take care of himself; and even the philosopher will manage to filter enough of homely reality out of his theorisings to place him at a certain advantage in the struggle for survival. The unscientific and the unphilosophical, on the other hand, are mostly at the mercy of every ill wind that blows. They stand or walk in the midst of the mysterious forces that surround them like children in the darkness of the night. They see nothing clearly, but they fear everything: and seeing nothing as it really is, they are unable to provide against danger, or even to deliver themselves from the childish helplessness of irrational panic.

At the present time, when cholera is prevailing, and is likely to prevail more, a little knowledge of the history of epidemics is both fortifying and consoling. One fact emerges large and reassuring as we study the history of European epidemics during the last five or six hundred years: it is this; that whereas six hundred years ago epidemics conquered and almost annihilated some of the most civilised nations of the world, at the present time the nations which can boast of intelligent sanitation on a large and practical scale are everywhere conquering and annihilating some of the most dreaded epidemics. Let us take two brief historical periods and set them over against each other! About the middle of the fourteenth century what was known as the "Black Death" visited almost every part of Asia, Northern Africa, and Europe, including Great Britain. The ravages of that epidemic, as reported by the various chroniclers, are almost impossible of belief. In the town of Cairo, for example, from ten to fifteen thousand people died every day. In China, alone, more than thirteen millions were carried off. India, it is declared, was "depopulated." "Tartary, the Tartar kingdom of Kaptschak, Mesopotamia, Syria, and Armenia were covered with dead bodies . . . in Caramania and Cæsarea none were left alive . . . Cyprus lost almost all its inhabitants; and ships without crews were often seen in the Mediterranean, as afterwards in the North Sea, driving about, and spreading the plague wherever they went on shore . . . The plague spread over England with unexampled rapidity, after it had first broken out in the county of Dorset, whence it advanced through the counties of Devon and Somerset to Bristol, and thence reached Gloucester, Oxford, and London. Probably few places escaped, perhaps not any; for the annals of contemporaries report that throughout the land only a tenth part of the inhabitants remained alive." As in England, so on the Continent. "At Avignon the Pope found it necessary to consecrate the Rhone, that bodies might be thrown into the river without delay, as the churchyards would no longer hold them."

Such are the facts which any reader may gather from Hecker's "Epidemics of the Middle Ages" and similar records. Now let us contrast them with a portion of the epidemic history of our own times. In 1817 Cholera spread rapidly throughout Bengal, extending during the following year over the greater part of Hindustan, and from thence to Ceylon, Burmah, and China. The disease travelled northward, but did not extend into Europe. In 1840-41, and until 1843-44 another epidemic prevailed, which, commencing in India, spread over the greater part of Asia and Europe, and finally reached America. In 1864-5 a fresh outbreak occurred, and this again spread through Asia and Europe, and finally spent itself in the United States. Since that time several epidemics have been experienced, but the efficacy of sanitation in checking their ravages has constantly become more and more manifest. So much has this been the case that Mr. (now Sir John) Simon felt justified, not many years ago, in writing thus: "Infinitely the most important preventive measures to be adopted against Cholera are to provide a pure supply of drinking water, good drainage, ventilation, and cleanliness, for these means, if rightly enforced, must prevent the Cholera contagion, whether disinfected or not, from acting to any great extent on the population."

The history of the last twenty years abundantly confirms Sir John Simon's judgment. There have been epidemics which have shown remarkable virulence in unsanitary localities; but so effective has even moderately good sanitation shown itself to be, that there has been witnessed many a time in the very same towns almost complete immunity in the wholesome quarters at the very time when the filthy streets and lanes were being decimated by the most deadly disease. On several occasions within quite recent recollection cholera itself has rapidly spread along the routes of travel from Asia through Europe, and boldly approached our own shores. But on each occasion our very partial sanitation and the promptness with which we have employed preventive measures have saved us from its terrible ravages.

The least intelligent reader of the daily papers cannot but be struck at the present moment with this remarkable fact, that those who know most about cholera decidedly fear it the least. Not a single medical officer at any of our ports appears to have so much as thought of running away from his duty. On the contrary, every man of them, from the greyest senior to the rawest assistant, has stuck manfully to his duties, and worked both day and night. This is a most reassuring spectacle, for, brave as doctors are by nature and training, it cannot but be that here and there a man of faint heart will be found among them. Yet even the faint-hearted are so convinced of the power of medical and sanitary science to keep the dread enemy in check that they stand manfully to their guns with the bravest. Our people, notwithstanding the alarming contents of the papers from day to day, have as yet shown no sign of panic. Let them continue to preserve this rational frame of mind, and to resolutely carry out the preventing instructions which they receive, and it will be strange, indeed, if they do not have their reward in the effectual checking of the dangerous and deadly epidemic which now threatens them.

